# Who gets help? Analyzing disparities in diagnosis, treatment, and care seeking of anxiety and depression among women in Bangladesh

**DOI:** 10.1371/journal.pmen.0000620

**Published:** 2026-05-20

**Authors:** Isna Haque Sheoti, Md. Zakiul Alam

**Affiliations:** 1 Department of Population Sciences, University of Dhaka, Dhaka, ‌‌Bangladesh; 2 Department of Population, Family and Reproductive Health, Johns Hopkins Bloomberg School of Public Health, Johns Hopkins University, Baltimore, ‌‌Maryland, United States of America; PLOS: Public Library of Science, UNITED KINGDOM OF GREAT BRITAIN AND NORTHERN IRELAND

## Abstract

Reproductive-aged women in Bangladesh exhibit low rates of anxiety and depression diagnosis, treatment, and care-seeking. This study investigated these factors and their correlates. Using data from the 2022 Bangladesh Demographic and Health Survey, we analyzed diagnosis (whether a healthcare provider ever informed), treatment (prescribed medication), and care-seeking (ever sought help and source). Log-binomial and Poisson regression models identified associated factors. Overall, 7.6% of respondents reported ever being diagnosed with anxiety and/or depression. When disaggregated, 3.39% were diagnosed with anxiety only, 0.56% with depression only, and 3.62% with both conditions. Furthermore, 11.85% had sought care from the formal sector. Compared with women aged 15–19 years, those aged 25 years and above showed significantly higher prevalence of diagnosis and care-seeking, while treatment was significantly higher among women aged 40–49 years. After adjustment, regional and residence-based differences in treatment were not statistically significant; however, some divisions showed significantly higher odds of care-seeking. Women with a positive attitude towards wife beating showed higher levels of diagnosis (PR = 1.28, CI: 1.06, 1.45, p = 0.008), treatment (PR = 1.50, CI: 1.17, 1.92, p = 0.001), and care-seeking (PR = 1.52, CI: 1.34, 1.72, p < 0.001) but 37% lower formal care-seeking (PR: 0.63, 95% CI: 0.43, 0.91, p = 0.013). Middle class (PR = 1.19, CI: 1.00, 1.41, p = 0.046) and working women (PR = 1.22, CI: 1.09, 1.38, p = 0.001) had a higher care-seeking, while non-internet users (PR = 0.67, CI: 0.59, 0.77, p < 0.001) had a lower prevalence of care-seeking. Women with a bank account were 42% more likely to seek formal care (PR: 1.42, 95% CI: 1.06, 1.91, p = 0.019). Policy actions are needed to reduce mental health stigma and establish specialized departments in government hospitals. Efforts to improve mental health care utilization should focus on enhancing financial access, expanding internet use, and addressing women’s attitudes that influence care-seeking behavior.

## Introduction

Anxiety and depression are the most common global mental health disorders, affecting around 4% and 5% of the world’s population, respectively [1,2]. Despite its higher prevalence, there is a persistent gap between the prevalence of these disorders and the rates of their diagnosis, treatment, and care-seeking, particularly in low-income countries [3,4]. Even in higher-income settings, only 25% of people with anxiety problems receive appropriate treatment.[3–5] This treatment gap is particularly pronounced for women who report higher rates of anxiety and depression compared to men [1,2]. In Bangladesh, recent data indicate that around 20% of reproductive-aged women face anxiety, and 5% of women face depression [6].

There are many treatment options available for anxiety disorders. Selective serotonin reuptake inhibitors and cognitive-behavioral therapy were found to be effective in treating anxiety [7]. Various scientific studies often suggest a combination of psychological therapy and pharmacotherapy [8]. Similarly, there are several treatment options available for depression that can help lessen the symptoms and severity of depression [9]. One option is psychotherapy, which is also called therapy or counseling, prioritizing thoughts, issues in life, and feelings of the patient [9]. Alternatively, there is a scope of treatment with medications commonly known as antidepressants [9]. Healthcare professionals often suggest lifestyle changes simultaneously with therapy for treating both anxiety and depression [8–10].

If left undiagnosed and untreated, anxiety and depression carry significant consequences at both individual and societal levels [11,12]. Untreated mental health issues are linked to reduced productivity, disability, heightened risk of self-harm and suicide, and overall a decreased quality of life. [11–13]. The economic toll of these disorders is substantial, often mirroring that of other major diseases like dementia in high-income regions [11]. For reproductive-aged women, the untreated anxiety and depression pose distinct maternal and perinatal risks, including a surging risk of preterm birth, spontaneous abortion, perinatal depression, and low birth weight. [14,15].

Addressing this public health burden requires a comprehensive understanding of the care continuum: from symptom experience to diagnosis, treatment initiation, and help-seeking behavior. Prior studies in Bangladesh have examined admission and discharge patterns, motivation and barriers to care-seeking, care-seeking behavior, the treatment gap among the adult population, cancer patients, the COVID-19 situation, and the rural population [16–20]. Recent analyses of nationally representative data have provided important insights into women’s mental health help-seeking behavior in Bangladesh, particularly in relation to early help-seeking and empowerment. However, the existing evidence remains limited in scope, as it does not address the full continuum of care, including diagnosis, treatment, and distinctions between formal and informal care-seeking [21–24]. Under these circumstances, our study aimed to explore the situation of diagnosis, treatment, and care-seeking behavior of anxiety and depression, and factors associated with them among reproductive-aged women (aged 15–49) using the 2022 Bangladesh Demographic and Health Survey (BDHS) data.

### Data and methods

#### Data source.

**Fig 1:**
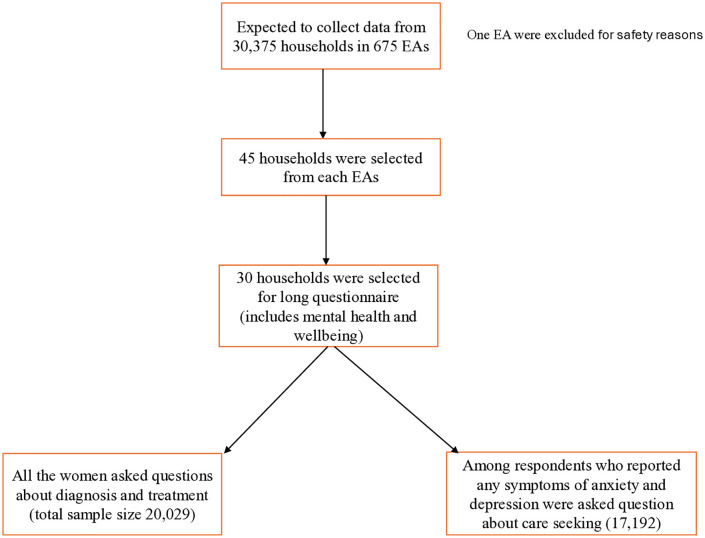
Sample selection process for the measurement of diagnosis, treatment, and care-seeking of anxiety and depression.

This study used data from BDHS-2022, a nationally representative cross-sectional survey [6]. The detailed methodology is available elsewhere [6]. The BDHS 2022 collected data on the mental health and well-being of reproductive-age women using a two-stage stratified cluster sampling. Initially, targeting 30,375 households across 675 enumeration areas (EAs), one EA in Cox’s Bazar was excluded for safety reasons, leaving 674 for data collection (**Fig 1**). From 45 households in each EA, 30 were selected for a long questionnaire that included a mental health and well-being section. Of 20,029 women surveyed about diagnosis and treatment, 17,192 who reported any symptoms of anxiety and/or depression in the preceding two weeks were questioned about care-seeking.

### Variables of study

#### Dependent variables.

This study examined four main dependent variables: diagnosis, treatment, care-seeking, and source of care-seeking of anxiety and depression as measured by BDHS in the long questionnaire. Diagnosis was defined as ‘percentage of ever-married women aged 15–49 who have ever been told by a health care provider that they have anxiety or depression’. Treatment involved taking prescribed medicine for these conditions in the two weeks before the survey. Care-seeking was identified as ‘among women with any symptoms of anxiety or depression in the 2 weeks preceding the survey, the percentage who have ever sought help’. Symptomatic respondents scored ‘1’ or higher on at least one item of the Generalized Anxiety Disorder 7-item scale (GAD-7) or the Patient Health Questionnaire 9-item scale (PHQ-9) scales; those scoring ‘0’ on all items were excluded from the care-seeking analysis [6]. To distinguish between professional and community-based support, a binary variable, Formal versus Informal Care-Seeking, was created. This variable categorizes help-seeking behaviors for anxiety/depression, based on the survey question: “From whom have you sought help?” Formal care includes seeking assistance from professionals, including doctors/ medical personnel, social service organizations, social workers, community health workers/fieldworkers, and non-governmental organizations (NGOs). Informal care involved seeking help from personal or community sources (e.g., spouse, family, friends, religious leaders, or others) when no formal care was pursued [25–27].

#### Independent variables.

For variable selection, we used the ‘A‌‌ndersen Behavioral Model of Health Services Use’ developed by Ronald M. Anderson [28–30], the existing literature [21–24], and the availability of variables in data [6]. The Andersen model explains why individuals and communities use or fail to use health services.[30,31] It implies that health service use is an effect of the interplay between personal, social, and systematic factors [28]. The framework suggests that three categories of factors determine utilization of health services: predisposing factors such as age, gender, education, or health beliefs; enabling factors such as income, insurance coverage, or the availability of medical facilities; and need factors such as care-seeking [28–32].

The final independent variables employed in this study are age, place of residence, division, wealth index, highest educational level, working status, participation in decision-making, having an account in a bank or other financial institution, religion, attitudes toward wife beating, husband’s level of education, exposure to any mass media, and ever used the internet (Table 1). BDHS measured the wealth index, an indicator of socioeconomic status, using principal component analysis and divided it into five economic quintiles. Exposure to mass media was defined by whether women had access to any TV, radio, or newspaper. Women’s participation in household decision-making was measured using four BDHS questions regarding decisions on large household purchases, family or relatives’ visits, household expenditures of earnings, and the respondent’s health care. If all the decisions were made by the respondent alone or together with the husband, it was defined as ‘yes,’ and if all the decisions were made by the husband alone or others, it was described as ‘no.’ Attitude towards wife beating was ‘positive,’ if respondents justified beating for any of these reasons: child neglects, leaving home without telling husband, refusing ‌‌sex, or arguing with husband; otherwise, it was ‘no’ if they responded negatively to all items.

**Table 1:** Sample characteristics by diagnosis, treatment, and care-seeking of anxiety / depression.

Variables	Diagnosis ^a^			Treatment ^a^			Care-seeking ^a^		
	**No** (**N** = 18,511)	**Yes** (**N** = 1,518)	**p-value**	**No** (**N** = 19,621)	**Yes** (**N** = 407)	**p-value**	**No** (**N** = 15,149)	**Yes** (**N** = 2,043)	**p-value**
**Age in 5-year groups**			<0.001			<0.001			0.002
15-19	1,653 (8.9)	76 (5.0)		1,704 (8.7)	25 (6.1)		1,215 (8.0)	121 (5.9)	
20-24	3,131 (16.9)	159 (10.5)		3,257 (16.6)	33 (8.0)		2,425 (16.0)	273 (13.4)	
25-29	3,262 (17.6)	261 (17.2)		3,459 (17.6)	65 (15.8)		2,637 (17.4)	346 (16.9)	
30-34	3,198 (17.3)	239 (15.7)		3,371 (17.2)	66 (16.1)		2,651 (17.5)	367 (18.0)	
35-39	3,046 (16.5)	299 (19.7)		3,266 (16.6)	79 (19.3)		2,557 (16.9)	388 (19.0)	
40-44	2,302 (12.4)	244 (16.1)		2,469 (12.6)	77 (18.9)		1,979 (13.1)	290 (14.2)	
45-49	1,918 (10.4)	241 (15.9)		2,095 (10.7)	64 (15.7)		1,684 (11.1)	258 (12.6)	
**Mean (SD)**	31.64 (8.91)	34.35 (8.75)	<0.001	31.79 (8.93)	34.70 (8.64)	<0.001	32.08 (8.90)	33.19 (8.69)	<0.001
**Division**			0.007			0.007			<0.001
Barishal	1,122 (6.1)	77 (5.1)		1,173 (6.0)	26 (6.4)		1,044 (6.9)	66 (3.2)	<0.001
Chattogram	3,414 (18.4)	335 (22.1)		3,643 (18.6)	106 (26.0)		2,756 (18.2)	445 (21.8)	
Dhaka	4,799 (25.9)	281 (18.5)		4,996 (25.5)	84 (20.7)		3,869 (25.5)	460 (22.5)	
Khulna	2,155 (11.6)	233 (15.4)		2,357 (12.0)	31 (7.7)		1,742 (11.5)	315 (15.4)	
Mymensingh	1,459 (7.9)	68 (4.5)		1,496 (7.6)	31 (7.6)		1,097 (7.2)	117 (5.7)	
Rajshahi	2,330 (12.6)	295 (19.4)		2,557 (13.0)	68 (16.8)		1,924 (12.7)	304 (14.9)	
Rangpur	2,131 (11.5)	159 (10.5)		2,255 (11.5)	36 (8.8)		1,768 (11.7)	241 (11.8)	
Sylhet	1,100 (5.9)	69 (4.5)		1,144 (5.8)	25 (6.0)		950 (6.3)	95 (4.6)	
**Type of place of residence**			0.472			0.187			0.295
Urban	5,293 (28.6)	407 (26.8)		5,600 (28.5)	100 (24.6)		4,307 (28.4)	540 (26.5)	
Rural	13,217 (71.4)	1,111 (73.2)		14,021 (71.5)	307 (75.4)		10,842 (71.6)	1,502 (73.5)	
**Highest educational level**			0.011			0.504			0.007
No education	2,503 (13.5)	250 (16.4)		2,692 (13.7)	61 (14.9)		2,237 (14.8)	241 (11.8)	
Primary	4,796 (25.9)	418 (27.5)		5,097 (26.0)	117 (28.7)		4,061 (26.8)	527 (25.8)	
Secondary	8,676 (46.9)	682 (45.0)		9,177 (46.8)	182 (44.6)		6,904 (45.6)	996 (48.8)	
Higher	2,535 (13.7)	168 (11.1)		2,655 (13.5)	48 (11.8)		1,948 (12.9)	279 (13.6)	
**Mean (SD)**	6.758 (4.193)	6.218 (4.225)	<0.001	6.723 (4.195)	6.394 (4.320)	0.218	6.554 (4.203)	6.977 (4.172)	<0.001
**Wealth of the household**			0.232			0.478			0.399
Poorest	3,336 (18.0)	247 (16.3)		3,518 (17.9)	65 (15.9)		2,810 (18.6)	342 (16.8)	
Poorer	3,686 (19.9)	341 (22.5)		3,944 (20.1)	83 (20.4)		3,095 (20.4)	399 (19.6)	
Middle	3,799 (20.5)	337 (22.2)		4,062 (20.7)	74 (18.1)		3,097 (20.4)	451 (22.1)	
Richer	3,891 (21.0)	298 (19.6)		4,098 (20.9)	91 (22.3)		3,141 (20.7)	430 (21.0)	
Richest	3,799 (20.5)	295 (19.4)		3,999 (20.4)	95 (23.3)		3,006 (19.8)	420 (20.6)	
**Respondent currently working**			0.128			0.606			<0.001
No	12,619 (68.2)	998 (65.7)		13,334 (68.0)	283 (69.4)		10,327 (68.2)	1,266 (62.0)	
Yes	5,891 (31.8)	520 (34.3)		6,287 (32.0)	125 (30.6)		4,822 (31.8)	776 (38.0)	
**Account in a bank or other financial institution**			0.010			<0.001			<0.001
No	14,865 (80.3)	1,164 (76.7)		15,734 (80.2)	295 (72.5)		12,173 (80.4)	1,540 (75.4)	
Yes	3,645 (19.7)	354 (23.3)		3,887 (19.8)	112 (27.5)		2,976 (19.6)	503 (24.6)	
**Participation in decision-making**			0.099			0.248			0.780
No	5,691 (30.7)	428 (28.2)		5,983 (30.5)	136 (33.4)		4,615 (30.5)	615 (30.1)	
Yes	12,820 (69.3)	1,090 (71.8)		13,638 (69.5)	271 (66.6)		10,534 (69.5)	1,428 (69.9)	
**Attitudes toward wife beating**			<0.001			<0.001			<0.001
Yes	2,820 (15.2)	295 (19.4)		3,027 (15.4)	88 (21.7)		2,321 (15.3)	460 (22.5)	
No	15,691 (84.8)	1,223 (80.6)		16,594 (84.6)	319 (78.3)		12,828 (84.7)	1,583 (77.5)	
**Husband’s level of education** ^**b**^			<0.001			0.303			0.052
No education	3,885 (21.0)	401 (26.4)		4,189 (21.3)	98 (23.9)		3,391 (22.4)	420 (20.6)	
Primary	5,268 (28.5)	400 (26.4)		5,571 (28.4)	98 (24.1)		4,346 (28.7)	565 (27.7)	
Secondary	6,067 (32.8)	472 (31.1)		6,397 (32.6)	142 (34.8)		4,876 (32.2)	663 (32.5)	
Higher	3,290 (17.8)	245 (16.1)		3,465 (17.7)	70 (17.2)		2,535 (16.7)	394 (19.3)	
**Religion**			0.101			0.584			0.859
Others	1,807 (9.8)	114 (7.5)		1,877 (9.6)	45 (10.9)		1,431 (9.4)	188 (9.2)	
Muslim	16,703 (90.2)	1,404 (92.5)		17,744 (90.4)	363 (89.1)		13,718 (90.6)	1,854 (90.8)	
**Exposed to any mass media**			0.666			0.520			0.016
No	7,781 (42.0)	651 (42.9)		8,268 (42.1)	164 (40.2)		6,452 (42.6)	795 (38.9)	
Yes	10,729 (58.0)	867 (57.1)		11,353 (57.9)	244 (59.8)		8,697 (57.4)	1,248 (61.1)	
**Ever used internet**			0.155			0.856			<0.001
No	12,904 (69.7)	1,096 (72.2)		13,717 (69.9)	283 (69.4)		10,841 (71.6)	1,296 (63.5)	
Yes	5,607 (30.3)	422 (27.8)		5,904 (30.1)	125 (30.6)		4,308 (28.4)	746 (36.5)	
**Total Sample**	20,029			20,029			17,192		

a: number (percentage). Percentage in parentheses.

b: 33 respondents answered, ‘don’t know’, and 1000 responses were missing. We imputed this using the simple Hot Deck imputation method.

Note: Percentages are column percentages representing the distribution of respondents across outcome categories, not subgroup-specific prevalence.

### Statistical analysis

We analyzed sample characteristics by outcomes (diagnosis, treatment, and care-seeking) using column percentages to describe group distributions (Table 1). Rao and Scott’s corrected Chi-square test was employed to assess associations within the complex survey design [33]. To identify factors associated with outcomes, we utilized log-binomial regression to estimate prevalence ratios (PRs). This approach was chosen over logistic regression because PRs offer more direct interpretability in cross-sectional studies and avoid the potential overestimation of associations inherent in Odds Ratios (ORs) when outcomes are not rare [34]. All models (adjusted and unadjusted) were run for all outcomes except the source of care. For formal vs informal sources of care-seeking, we used Poisson regression because the log-binomial regression failed to converge. Due to convergence issues with the log-binomial model, a Poisson regression model with a log link was fitted to estimate prevalence ratios. For models that failed to converge (specifically, formal vs. informal source of care), we employed modified Poisson regression. All models were executed using the svy prefix in Stata 18, which utilizes Taylor-series linearization to provide robust (sandwich) variance estimators, ensuring consistent standard errors and 95% Confidence Intervals (CIs).

Model diagnostics were rigorously evaluated. Multicollinearity was ruled out using Variance Inflation Factors (VIFs) in parallel weighted linear models; across all outcomes, the maximum observed VIF was 4.07 (in the Formal vs. Informal Care model for the region variable), well below the conservative threshold of 5.0. Model specification and goodness-of-fit were evaluated using the Pregibon Link Test. In this large-scale survey analysis, the appropriateness of the log-link function and covariate structure was confirmed by assessing the significance of the squared prediction variable. While the squared prediction variable reached statistical significance across the outcomes (Diagnosis: p = 0.001; Treatment: p = 0.011; Care-seeking: p < 0.001), this was interpreted as reflecting high statistical power in our large sample (unweighted N = 17,243–19,987) rather than clinically meaningful misspecification. The consistency of results across log-binomial and modified Poisson frameworks further supported model stability.

The analysis accounted for the complex survey design by incorporating the primary sampling units [v001], the Stratification variable [v022], and the sampling weights [v005]. Missing data handling for husband’s education (unweighted n = 1,033 “don’t know” or missing) was addressed through sequential (simple) hot-deck imputation. To ensure the stability of our inferences, we performed a sensitivity analysis using complete-case analysis. Sensitivity analyses demonstrated high concordance between the imputed and complete-case models across all outcome measures, with the PR for ‘no husband education’ remaining stable for diagnosis (1.08 [0.87–1.35] vs. 1.17 [0.92–1.50]), treatment (1.00 [0.64–1.57] vs. 1.07 [0.64–1.78]), and care-seeking (0.84 [0.70–1.02] vs. 0.84 [0.68–1.02]). The consistency of the PRs across both the imputed and complete-case models confirms that the handling of missing data did not bias the final results.

### Ethics statement

Our study used secondary data from the Bangladesh Demographic and Health Survey 2022, negating the need for separate IRB approval. The BDHS survey, a collaborative effort by the Ministry of Health and Family Welfare, the National Institute of Population Research and Training (NIPORT), Mitra and Associates, and ICF International, obtained verbal informed consent using an informed consent statement and ethical approval from the Bangladesh Medical Research Council. The dataset is available at https://dhsprogram.com/data, and full ethics are in the BDHS report [6].

## Results

### Background characteristics

Table 1 presents the distribution of sample characteristics by diagnosis, treatment, and care-seeking of anxiety and depression among respondents. Among those who received a diagnosis, treatment, and care-seeking, a relatively larger proportion were aged 35–39 years (19.7%, 19.3%, and 19.0%, respectively), reflecting the age distribution of the study population. Similarly, the largest share of respondents with the diagnosis (45.0%), treatment (44.6%), and care-seeking (48.8%) had a secondary level of education. In terms of employment and attitudes, a greater proportion of respondents who received diagnosis, treatment, and care-seeking were not currently working and did not justify wife beating. Additionally, the majority of respondents with a diagnosis (92.5%), treatment (89.1%), and care-seeking (90.8%) were Muslim, consistent with the overall religious composition of the sample.

### Prevalence of diagnosis, treatment, and care-seeking for anxiety and/or depression

**Fig 2:**
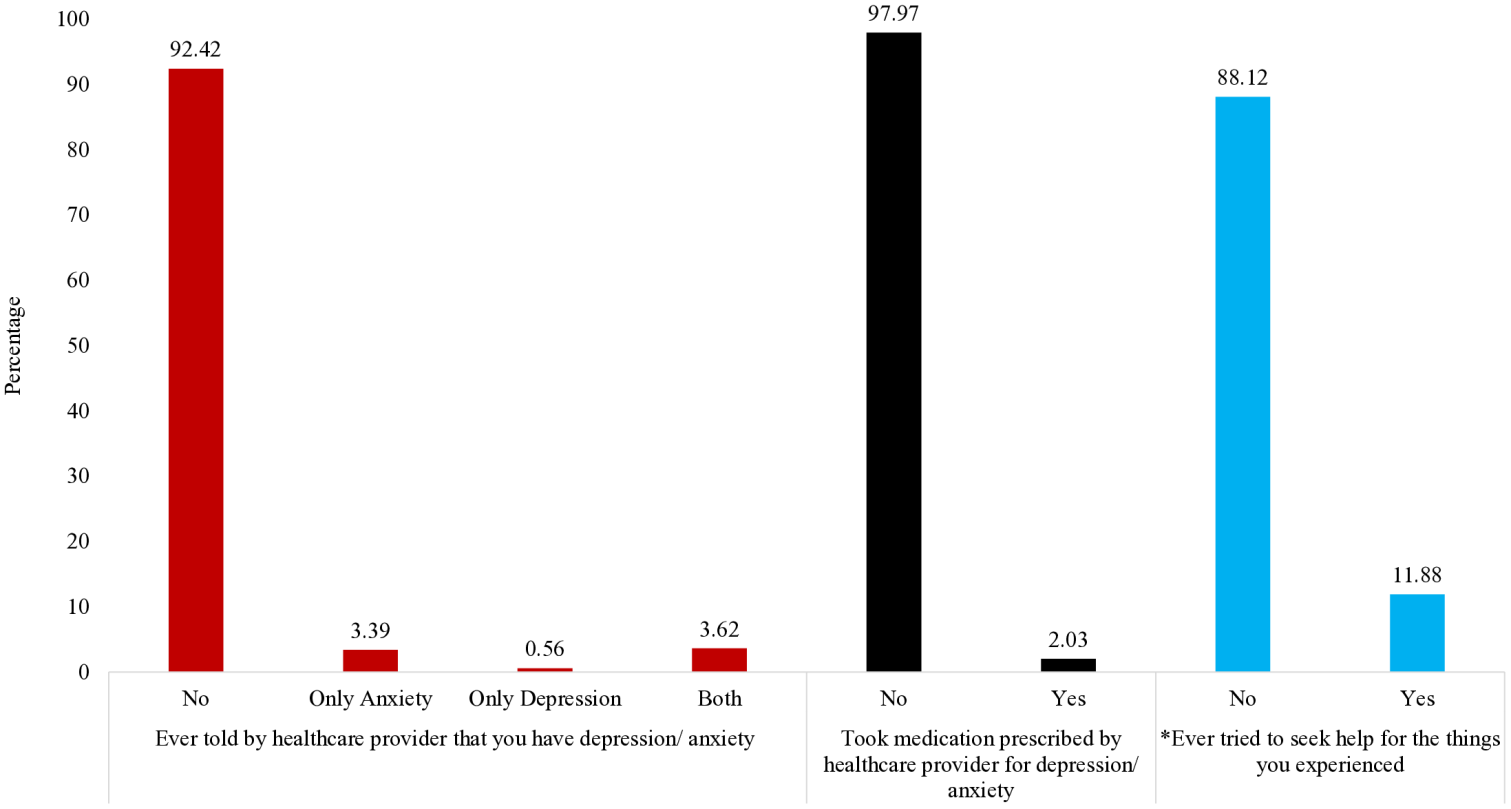
Situation of Diagnosis, treatment, and care-seeking of anxiety and depression (n = 17,192). *Percentages are calculated among respondents classified as symptomatic. A respondent was defined as symptomatic if they scored ‘1’ (several days/rarely), ‘2’ (more than half the days/often), or ‘3’ (nearly every day/always) on at least one item of the Generalized Anxiety Disorder 7-item scale (GAD-7) or the Patient Health Questionnaire 9-item scale (PHQ-9). Respondents scoring ‘0’ (not at all/never) on all items were excluded [6]. Fig 2 presents mutually exclusive categories derived from two indicators: “Ever diagnosed with anxiety” (7.0%) and “Ever diagnosed with depression” (4.2%). Individuals reporting both conditions were categorized into a combined “Anxiety and/or Depression” group to ensure mutual exclusivity.

**Fig 2** presents the situation of diagnosis and treatment of anxiety and depression among respondents. Only 2.03% of respondents received medication or treatment provided by a doctor or healthcare worker. In the case of diagnosis, 3.39% of respondents were diagnosed with anxiety, 0.56% were diagnosed with depression, and 3.62% were diagnosed with both by a provider. Only 11.88% of women who experienced symptoms (1–3 on the depression and/or anxiety scale) ever tried to seek any form of help.

### Diagnosis of anxiety and/or depression

**Table 2** presents factors associated with the diagnosis of anxiety and depression among participants using log-binomial regression. Women aged 25 or older had significant differences with women aged 15–19 in the prevalence of diagnosis. Compared to women aged 15–19, the prevalence of diagnosis was significantly higher among those aged 25–29 (PR:1.74, CI: 1.29, 2.35, p < 0.001), 30–34 (PR:1.60, CI: 1.18, 2.18, p = 0.003), 35–39 (PR:2.03, CI: 1.52, 2.71, p < 0.001), 40–44 (PR:2.18, CI: 1.59, 2.99, p < 0.001), and 45–49 (PR = 2.52, CI: 1.86, 3.42, p < 0.001). Women living in Khulna and Rajshahi had significantly higher prevalence of diagnosis of anxiety/depression than women living in Mymensingh. Lastly, women with a positive attitude towards wife beating had significantly higher levels of diagnosis (PR = 1.28, CI: 1.06, 1.45, p = 0.008) than women with a negative attitude.

**Table 2:** Factors associated with diagnosis of anxiety and depression (n = 20,029).

	Unadjusted model		Adjusted model	
**Variables**	**Prevalence ratio [95% CI]**	**p-value**	**Prevalence ratio [95% CI]**	**p-value**
**Age of the respondent**				
20-24	1.09[0.82,1.46]	0.524	1.13[0.84,1.51]	0.410
25-29	1.69[1.26,2.26]	<0.001	1.74[1.29,2.35]	<0.001
30-34	1.58[1.18,2.13]	0.002	1.60[1.18,2.18]	0.003
35-39	2.03[1.54,2.68]	<0.001	2.03[1.52,2.71]	<0.001
40-44	2.18[1.62,2.93]	<0.001	2.18[1.59,2.99]	<0.001
45-49	2.54[1.90,3.40]	<0.001	2.52[1.86,3.42]	<0.001
15-19(RC)	1		1	
**Division**				
Barishal	1.43[0.96,2.15]	0.082	1.44[0.96,2.15]	0.078
Chattogram	1.99[1.29,3.08]	0.002	2.04[1.29,3.22]	0.002
Dhaka	1.24[0.84,1.82]	0.282	1.25[0.85,1.85]	0.258
Khulna	2.18[1.51,3.15]	<0.001	2.17[1.49,3.16]	<0.001
Rajshahi	2.51[1.71,3.68]	<0.001	2.44[1.66,3.58]	<0.001
Rangpur	1.55[1.02,2.36]	0.039	1.61 [1.06, 2.45]	0.025
Sylhet	1.31[0.89,1.93]	0.167	1.36[0.92,2.00]	0.122
Mymensingh (RC)	1		1	
**Place of residence**				
Rural	1.09[0.87,1.36]	0.473	0.98[0.78,1.22]	0.842
Urban (RC)	1		1	
**Education of the respondent**				
No education	1.46[1.13,1.89]	0.004	1.05[1.78,1.43]	0.737
Primary	1.29[1.02,1.63]	0.031	1.11[0.87,1.41]	0.412
Secondary	1.17[0.95,1.44]	0.129	1.16[0.95,1.41]	0.153
Higher (RC)	1		1	
**Wealth**				
Poorest	0.96[0.73,1.25]	0.74	1.04[0.77,1.40]	0.812
Poorer	1.18[0.94,1.47]	0.16	1.18[0.93,1.50]	0.167
Middle	1.13[0.92,1.39]	0.243	1.12[0.90,1.39]	0.311
Richer	0.99[0.81,1.21]	0.897	1.00[0.82,1.22]	0.979
Richest (RC)	1		1	
**Working status**				
Yes	1.11[0.97,1.26]	0.127	0.96[0.83,1.12]	0.630
No (RC)	1		1	
**Has an account in a bank or other financial institution**				
Yes	1.22[1.05,1.41]	0.009	1.18[1.01,1.38]	0.042
No (RC)	1		1	
**Participation in decision-making**				
Yes	1.12[0.98,1.28]	0.099	1.07[0.93,1.22]	0.953
No (RC)	1		1	
**Attitude toward wife beating**				
Positive	1.31 [1.12,1.53]	0.001	1.24[1.06,1.45]	0.008
Negative (RC)	1		1	
**Husband’s level of education**				
No education	1.35 [1.09,1.67]	0.005	1.08[0.87,1.35]	0.480
Primary	1.02 [0.84,1.24]	0.838	0.91[0.74,1.12]	0.373
Secondary	1.04 [0.87,1.25]	0.652	0.98[0.82,1.16]	0.804
Higher (RC)	1		1	
**Religion**				
Muslim	1.31 [0.95,1.81]	0.105	1.35[0.97,1.87]	0.932
Others (RC)	1		1	
**Exposed to any mass media**				
Yes	0.97[0.84,1.12]	0.666	0.99[0.87,1.13]	0.932
No (RC)	1		1	
**Ever used internet**				
No	1.12[0.96,1.30]	0.156	0.99[0.84,1.19]	0.986
Yes (RC)	1		1	
RC = Reference category				

### Treatment of anxiety and/or depression

**Table 3** presents factors associated with the treatment of anxiety and depression among participants using log-binomial regression. Women in higher age groups had a higher prevalence ratio of getting treatment than younger age groups. Compared with other age groups, women aged 40–44 (PR:2.21, CI: 1.25, 3.91, p = 0.007) and 45–49 (PR:2.11, CI: 1.21, 3.68, p = 0.009) had significantly higher prevalence. Women living in Khulna (PR = 0.57, CI:0.36, 0.90, p = 0.017) had a significantly lower prevalence of treatment of anxiety and depression than women living in Mymensingh. Women who had a bank or other financial account (PR = 1.38, CI: 1.07, 1.79, p = 0.014) had a significantly higher prevalence of treatment than women without one. Lastly, women with a positive attitude towards wife beating had significantly higher levels of treatment (PR = 1.50, CI: 1.17,1.92, p = 0.001) than women with a negative attitude.

**Table 3:** Factors associated with treatment of anxiety and depression (n = 20,029).

	Unadjusted model		Adjusted model	
**Variables**	**Prevalence ratio [95% CI]**	**p-value**	**Prevalence ratio [95% CI]**	**p-value**
**Age of the respondent**				
20-24	0.69[0.37,1.30]	0.256	0.72[0.38,1.34]	0.299
25-29	1.28[0.74,2.20]	0.372	1.32[0.75,2.32]	0.330
30-34	1.33[0.75,2.37]	0.325	1.35[0.75,2.41]	0.316
35-39	1.65[0.94,2.88]	0.082	1.69[0.96,2.97]	0.070
40-44	2.11[1.22,3.64]	0.007	2.21[1.25,3.91]	0.007
45-49	2.07[1.19,3.60]	0.010	2.11[1.21,3.68]	0.009
15-19(RC)	1		1	
**Division**				
Barishal	1.07[0.69,1.69]	0.753	1.02[0.65,1.60]	0.942
Chattogram	1.40[0.90,2.19]	0.138	1.34[0.84,2.13]	0.222
Dhaka	0.82[0.51,1.31]	0.407	0.77[0.47,1.28]	0.320
Khulna	0.65[0.42,1.02]	0.062	0.57[0.36,0.90]	0.017
Rajshahi	1.29[0.85,1.96]	0.232	1.22[0.79,1.88]	0.359
Rangpur	0.78[0.49,1.24]	0.284	0.96 [0.57, 1.60]	0.312
Sylhet	1.04[0.63,1.73]	0.873	1.36[0.92,2.00]	0.864
Mymensingh (RC)	1		1	
**Place of residence**				
Rural	1.22[0.91,1.63]	0.188	1.33[0.93,1.90]	0.121
Urban (RC)	1		1	
**Education of the respondent**				
No education	1.24[0.81,1.90]	0.322	1.00[0.58,1.73]	0.999
Primary	1.26[0.87,1.82]	0.219	1.21[0.75,1.96]	0.441
Secondary	1.09[0.76,1.57]	0.636	1.12[0.74,1.70]	0.603
Higher (RC)	1		1	
**Wealth**				
Poorest	0.78[0.54,1.13]	0.188	0.80[0.49,1.30]	0.362
Poorer	0.89[0.65,1.23]	0.479	0.87[0.58,1.30]	0.490
Middle	0.77[0.55,1.07]	0.123	0.75[0.51,1.10]	0.136
Richer	0.94[0.68,1.30]	0.699	0.95[0.67,1.34]	0.757
Richest (RC)	1		1	
**Working status**				
Yes	0.93[0.73,1.21]	0.606	0.89[0.68,1.16]	0.385
No (RC)	1		1	
**Has an account in a bank or other financial institution**				
Yes	1.52[1.21,1.92]	<0.001	1.38[1.07,1.79]	0.014
No (RC)	1		1	
**Participation in decision-making**				
Yes	0.88[0.70,1.10]	0.248	0.82[0.65,1.02]	0.076
No (RC)	1		1	
**Attitude toward wife beating**				
Positive	1.51 [1.18,1.92]	0.001	1.50[1.17,1.92]	0.001
Negative (RC)	1		1	
**Husband’s level of education**				
No education	1.15 [0.82,1.61]	0.416	1.00[0.64,1.57]	0.996
Primary	0.87 [0.62,1.24]	0.448	0.84[0.52,1.34]	0.460
Secondary	1.10 [0.80,1.52]	0.582	1.06[0.71,1.58]	0.772
Higher (RC)	1		1	
**Religion**				
Muslim	0.86 [0.51,1.46]	0.584	0.87[0.51,1.48]	0.604
Others (RC)	1		1	
**Exposed to any mass media**				
Yes	1.08[0.85,1.37]	0.52	1.10[0.85,1.42]	0.487
No (RC)	1		1	
**Ever used internet**				
No	0.98[0.76,1.26]	0.856	0.97[0.70,1.34]	0.852
Yes (RC)	1		1	
RC = Reference category				

### Care-seeking of anxiety and/or depression

**Table 4** presents factors associated with care-seeking anxiety and depression among participants using log-binomial regression. Women aged 25 or older had significant differences with women aged 15–19 in their prevalence of care-seeking. Compared to women aged 15–19, the prevalence of care seeking was significantly higher among those aged 25–29 (PR:1.31, CI: 1.04, 1.66, p = 0.027), 30–34 (PR:1.40, CI: 1.10,1.77, p = 0.006), 35–39 (PR:1.61, CI: 1.26, 2.06, p < 0.001), 40–44 (PR:1.65, CI: 1.27, 2.14, p < 0.001), and 45–49 (PR = 1.85, CI: 1.37, 2.49, p < 0.001). Women living in Barishal (PR = 0.60, CI: 0.45, 0.81, p = 0.001) had a lower prevalence of care-seeking. However, women living in Chattogram (PR = 1.35, CI: 1.07, 1.72, p = 0.012), Khulna (PR = 1.45, CI: 1.17, 1.80, p = 0.001), and Rajshahi (PR = 1.27, CI: 1.00, 1.62, p = 0.048) had a significantly higher prevalence of care seeking for anxiety and/or depression than Mymensingh. Furthermore, women from middle-class households (PR = 1.19, CI: 1.00, 1.41, p = 0.046) had a significantly higher prevalence of seeking care than women of the richest wealth status. Women who were working (PR = 1.22, CI: 1.09, 1.38, p = 0.001) and had a positive attitude toward wife beating (PR = 1.52, CI: 1.34, 1.72, p < 0.001) received significantly higher care-seeking prevalence. Finally, women who didn’t use the internet had significantly lower care-seeking (PR = 0.67, CI: 0.59, 0.77, p < 0.001) than those who did.

**Table 4:** Factors associated with care-seeking for anxiety and depression (n = 17,192).

	Unadjusted model		Adjusted model	
**Variables**	**Prevalence ratio [95% CI]**	**p-value**	**Prevalence ratio [95% CI]**	**p-value**
**Age of the respondent**				
20-24	1.12[0.87,1.43]	0.372	1.14[0.89,1.46]	0.300
25-29	1.28[1.02,1.61]	0.034	1.31[1.04,1.66]	0.027
30-34	1.34[1.07,1.68]	0.01	1.40[1.10,1.77]	0.006
35-39	1.45[1.15,1.84]	0.002	1.61[1.26,2.06]	<0.001
40-44	1.41[1.12,1.78]	0.003	1.65[1.27,2.14]	<0.001
45-49	1.47[1.13,1.90]	0.004	1.85[1.37,2.49]	<0.001
15-19(RC)	1		1	
**Division**				
Barishal	0.62[0.46,0.84]	0.002	0.60[0.45,0.81]	0.001
Chattogram	1.45[1.15,1.82]	0.002	1.35[1.07,1.72]	0.012
Dhaka	1.10[0.86,1.42]	0.434	1.02[0.78,1.33]	0.893
Khulna	1.59[1.28,1.97]	<0.001	1.45[1.17,1.80]	0.001
Rajshahi	1.42[1.11,1.81]	0.005	1.27[1.00,1.62]	0.048
Rangpur	1.25[0.98,1.59]	0.07	1.23 [0.97, 1.57]	0.084
Sylhet	0.94[0.73,1.21]	0.639	0.96[0.75,1.23]	0.726
Mymensingh (RC)	1		1	
**Place of residence**				
Rural	1.09[0.93,1.29]	0.296	1.10[0.93,1.31]	0.255
Urban (RC)	1		1	
**Education of the respondent**				
No education	0.78[0.63,0.95]	0.015	0.80[0.61,1.04]	0.089
Primary	0.92[0.78,1.07]	0.287	1.01[0.83,1.24]	0.902
Secondary	1.01[0.87,1.17]	0.922	1.13[0.95,1.34]	0.178
Higher (RC)	1		1	
**Wealth**				
Poorest	0.89[0.74,1.07]	0.197	1.24[0.99,1.53]	0.051
Poorer	0.93[0.79,1.10]	0.415	1.14[0.95,1.38]	0.159
Middle	1.04[0.88,1.22]	0.672	1.19[1.00,1.41]	0.046
Richer	0.98[0.84,1.15]	0.808	1.10[0.94,1.28]	0.259
Richest (RC)	1		1	
**Working status**				
Yes	1.27[1.14,1.42]	<0.001	1.22[1.09,1.38]	0.001
No (RC)	1		1	
**Has an account in a bank or other financial institution**				
Yes	1.29[1.15,1.44]	<0.001	1.07[0.95,1.21]	0.263
No (RC)	1		1	
**Participation in decision-making**				
Yes	1.02[0.91,1.13]	0.78	0.96[0.86,1.07]	0.429
No (RC)	1		1	
**Attitude toward wife beating**				
Positive	1.51 [1.33,1.71]	<0.001	1.52[1.34,1.72]	<0.001
Negative (RC)	1		1	
**Husband’s level of education**				
No education	0.82 [0.70,0.96]	0.014	0.84[0.70,1.02]	0.074
Primary	0.86 [0.74,0.99]	0.033	0.87[0.74,1.03]	0.110
Secondary	0.89 [0.78,1.02]	0.09	0.88[0.76,1.03]	0.114
Higher (RC)	1		1	
**Religion**				
Muslim	1.02 [0.80,1.32]	0.859	1.05[0.81,1.36]	0.709
Others (RC)	1		1	
**Exposed to any mass media**				
Yes	1.15[1.02,1.28]	0.017	1.08[0.96,1.21]	0.210
No (RC)	1		1	
**Ever used internet**				
No	0.72[0.65,0.81]	<0.001	0.67[0.59,0.77]	<0.001
Yes (RC)	1		1	
RC = Reference category.				

### Source of care-seeking for anxiety and/or depression

**Fig 3:**
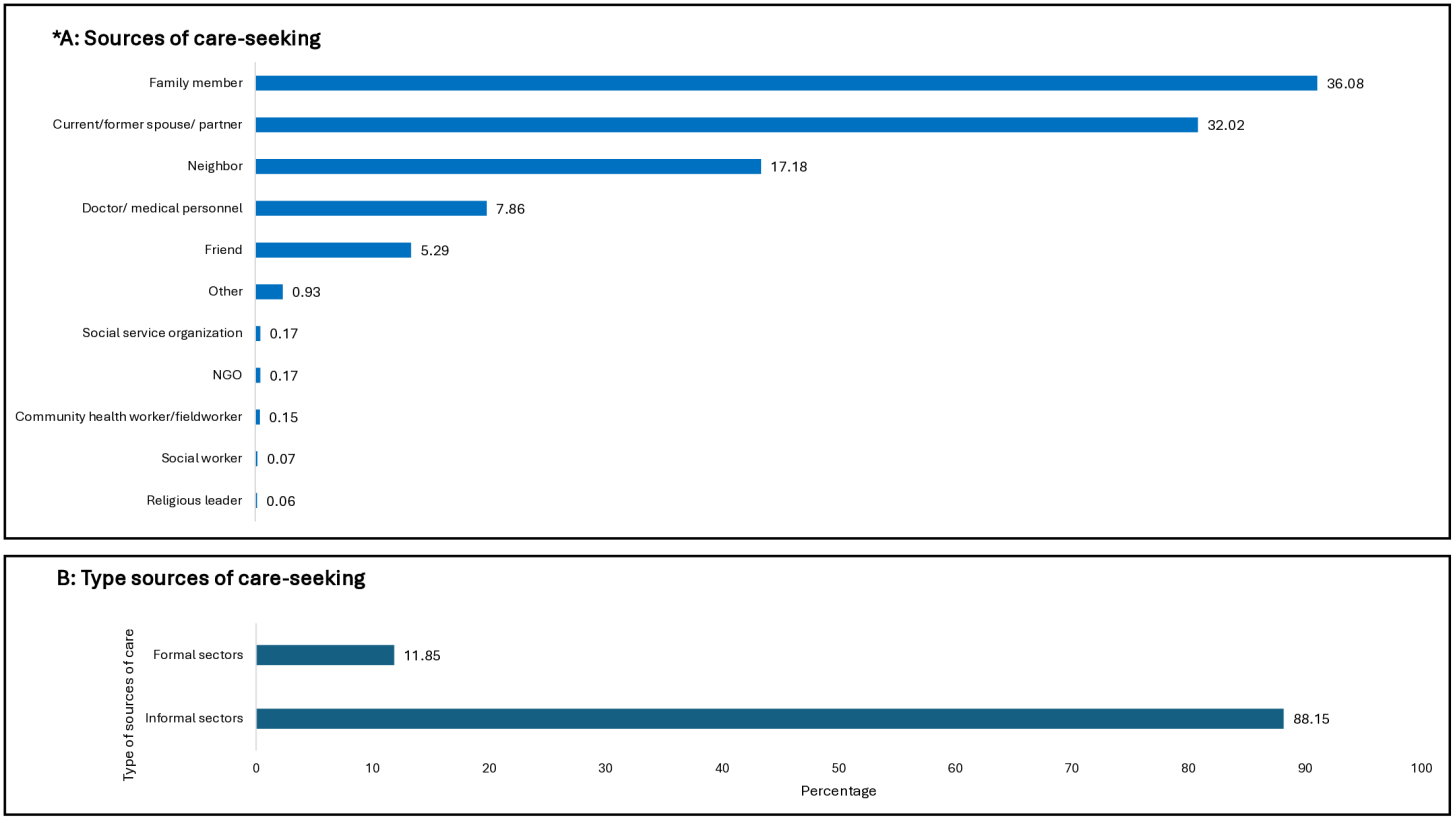
Source of care-seeking among respondents who ever sought health (n = 2043). * This Fig is based on multiple responses with 2001 valid cases (weighted). The percentage was produced using ‘percent of responses.’.

Fig 3 (A and B) presents the source of care-seeking among respondents. Most respondents sought care from their family members (36.08%), followed by current or former spouse or partner (32.02%). While 17.18% of respondents sought help from neighbors, only 7.86% sought help from doctors/medical personnel (Fig 3A). Only 11.85% had sought care from the formal sector (Fig 3B).

An analysis of multiple Poisson regression, for formal care-seeking (versus informal help) among symptomatic women, identified significant socioeconomic and demographic disparities (**Table 5**). The likelihood of accessing formal professional services increased considerably with age. Women aged 45–49 were nearly 5 times more likely to seek formal care (PR: 4.73, 95% CI: 1.73, 12.98, p = 0.003) than the youngest group (15–19 years). Women with a bank or other financial institution account were 42% more likely to seek formal care (PR: 1.42, 95% CI: 1.06, 1.91, p = 0.019). Women who expressed a positive attitude toward wife-beating had a 37% lower prevalence of seeking formal care (PR: 0.63, 95% CI: 0.43, 0.91, p = 0.013).

**Table 5:** Factors associated with formal care-seeking (vs informal) for anxiety and depression (n = 2043).

Variables	Unadjusted Model (Log-Binomial)		Adjusted Model (Poisson)	
**Prevalence Ratio [95% CI]**	**p-value**	**Prevalence Ratio [95% CI]**	**p-value**
**Age of the respondent**	(Ref: 15–19)		(Ref: 15–19)	
20-24	1.98 [0.71, 5.50]	0.189	2.00 [0.72, 5.53]	0.182
25-29	2.54 [0.90, 7.17]	0.079	2.62 [0.95, 7.27]	0.063
30-34	1.82 [0.62, 5.30]	0.275	1.78 [0.62, 5.10]	0.282
35-39	3.46 [1.22, 9.81]	0.019	3.26 [1.17, 9.14]	0.024
40-44	3.98 [1.42, 11.10]	0.009	4.14 [1.48, 11.60]	0.007
45-49	4.80 [1.72, 13.39]	0.003	4.73 [1.73, 12.98]	0.003
15-19(RC)				
**Division**				
Barishal	1.99 [0.85, 4.64]	0.111	1.84 [0.78, 4.32]	0.161
Chattogram	1.26 [0.62, 2.54]	0.521	1.23 [0.60, 2.55]	0.568
Dhaka	1.17 [0.55, 2.47]	0.683	1.05 [0.50, 2.21]	0.898
Khulna	0.68 [0.31, 1.50]	0.34	0.63 [0.29, 1.39]	0.249
Rajshahi	1.73 [0.82, 3.63]	0.148	1.76 [0.83, 3.72]	0.138
Rangpur	1.77 [0.84, 3.74]	0.136	1.77 [0.83, 3.78]	0.139
Sylhet	1.04 [0.43, 2.47]	0.937	0.89 [0.36, 2.20]	0.803
Mymensingh (RC)				
**Place of residence**				
Rural	0.97 [0.69, 1.36]	0.845	1.06 [0.75, 1.50]	0.753
Urban (RC)				
**Education of the respondent**				
No education	1.32 [0.81, 2.16]	0.262	0.88 [0.48, 1.63]	0.692
Primary	0.89 [0.58, 1.38]	0.613	0.75 [0.44, 1.28]	0.29
Secondary	1.06 [0.70, 1.59]	0.784	1.05 [0.67, 1.66]	0.831
Higher (RC)				
**Wealth**				
Poorest	0.73 [0.47, 1.15]	0.172	0.78 [0.45, 1.35]	0.369
Poorer	0.77 [0.50, 1.20]	0.248	0.77 [0.47, 1.26]	0.304
Middle	0.69 [0.44, 1.08]	0.102	0.69 [0.43, 1.12]	0.135
Richer	0.74 [0.49, 1.13]	0.166	0.76 [0.49, 1.18]	0.217
Richest (RC)				
**Working status**				
Yes	0.85 [0.63, 1.14]	0.275	0.82 [0.61, 1.11]	0.198
No (RC)				
**Has an account in a bank or other financial institution**				
Yes	1.42 [1.06, 1.91]	0.021	1.42 [1.06, 1.91]	0.019
No (RC)				
**Participation in decision-making**				
Yes	0.91 [0.69, 1.20]	0.52	0.80 [0.60, 1.07]	0.129
No (RC)				
**Attitude toward wife beating**				
Positive	0.59 [0.41, 0.85]	0.005	0.63 [0.43, 0.91]	0.013
Negative (RC)				
**Husband’s level of education**				
No education	1.09 [0.72, 1.66]	0.674	1.34 [0.78, 2.28]	0.289
Primary	0.94 [0.62, 1.42]	0.78	1.28 [0.80, 2.07]	0.302
Secondary	1.08 [0.72, 1.62]	0.713	1.31 [0.84, 2.05]	0.233
Higher (RC)				
**Religion**				
Muslim	1.10 [0.69, 1.76]	0.687	1.26 [0.77, 2.06]	0.359
Others (RC)				
**Exposed to any mass media**				
Yes	1.19 [0.88, 1.60]	0.264	1.21 [0.87, 1.67]	0.263
No (RC)				
**Ever used internet**				
No	1.32 [0.93, 1.88]	0.114	1.28 [0.82, 1.98]	0.273
Yes (RC)				

Note: Due to the inherent difficulty of the log-binomial model to converge (failed to converge) in multiple regression settings, particularly with complex survey data, the final model was estimated using Poisson regression.

## Discussion

This study explored the situation of diagnosis, treatment, and care-seeking of anxiety and depression and their determinants for reproductive-aged women in Bangladesh. Our key findings highlight a substantial unmet need: only 2.03% of respondents took medication or treatment from a doctor or health care worker, 3.39% were diagnosed with anxiety, and 0.56% were diagnosed with depression by the provider. Furthermore, while 11.88% of symptomatic women reported seeking some form of help, most of this care was derived from informal sources such as family members (36.08%) and spouses (32.02%), with only 7.86% seeking help from professionals (doctors/medical personnel). Our findings on diagnosis, treatment, and care-seeking differ from previous studies conducted in Bangladesh that reported a higher prevalence of anxiety and depression and comparatively higher levels of care-seeking among women [24,35,36]. For example, prior analyses using nationally representative data have documented a substantially greater burden of anxiety and depressive symptoms and higher engagement with care services [24,36]. These differences may be attributed to several methodological and definitional variations. First, our study defines care-seeking specifically among symptomatic individuals based on GAD-7 and PHQ-9 thresholds, whereas some previous studies considered broader populations or different cut-offs, potentially inflating prevalence estimates [24,35]. Second, our outcome definitions distinguish between diagnosis, treatment, and care-seeking, which may lead to lower observed estimates for formal treatment compared to studies that combine these outcomes [36]. Third, variations in analytical approaches, including the use of prevalence ratios and adjustment for multiple socioeconomic and behavioral factors, may further contribute to differences in reported estimates.[35] Finally, differences in the operationalization of help-seeking, particularly the distinction between formal and informal sources in our study, may explain the lower proportion of professional care-seeking observed [24,36].

The likelihood of accessing formal professional services increased considerably with age, indicating a substantial barrier for adolescents and young adults. Women having an account in a financial institution were more likely to seek formal care than non-account holders, suggesting that economic activity is a key determinant of access to professional services. This may be because financial inclusion enhances women’s economic autonomy, enabling them to afford consultation, transportation, and treatment costs [30,37]. It may also increase exposure to health information and awareness of mental health services [37].

Notably, the analysis revealed that adherence to adverse sociocultural norms acts as a structural barrier: women who expressed a positive attitude toward wife-beating had a lower prevalence of seeking formal care. It may reflect internalized patriarchal norms that reduce women’s autonomy and normalize suffering, thereby limiting disclosure and care-seeking for mental health conditions [21,38]. The diagnosis of mental disorders has been reported to be a stigmatized issue in studies around the world, with a lower rate of diagnosis and reluctance to it [39–43]. In Nepal, only 18.3% of women with any mental disorders (anxiety or depression) sought help [40], and in Ethiopia, 12.9% of women sought help, and 4.2% accessed the treatment [41]. Like our findings, women in Ethiopia sought most help from informal sources, especially husbands [41]. The higher help-seeking prevalence in Nepal may reflect recent national policies aimed at improving maternal mental health, including community-based screening programs, awareness campaigns, and integration of mental health services into primary healthcare [44,45]. Furthermore, a study of perinatal rural women in Bangladesh found that two-thirds of women didn’t seek any care during that critical period [17]. A survey of urban China presented a contradictory picture, with almost 82.2% of women seeking online support for their mental health [43]. This high uptake may reflect widespread digital literacy and acceptance of internet‑based mental health tools, which have been found to be acceptable and increasingly used in China’s mental health landscape, including via large online platforms facilitating psychological care delivery [46,47]. In contrast, digital mental health services in Bangladesh remain underdeveloped and face barriers related to stigma, limited mental health literacy, and lack of infrastructure, limiting uptake of online support there [48]. The Chinese experience suggests that expanding accessible digital mental health resources and awareness campaigns could be a promising strategy to improve service utilization among women in Bangladesh.

Women’s age, division of residence, and attitude towards wife beating were found to be significant determinants of the diagnosis of anxiety and depression. A study about perimenopausal women found higher vulnerability diagnoses of anxiety and depression among the older age group [49]. It is consistent with our research, as we saw higher age as a significant determinant of anxiety and depression. In this study, attitude toward wife beating was considered an indicator of gender norms and women’s empowerment rather than a direct measure of domestic violence, reflecting sociocultural contexts in which acceptance of such norms may be associated with greater vulnerability to adverse experiences and, consequently, higher engagement with mental health services. Our study found that a positive attitude towards wife beating is associated with significantly higher rates of diagnosis, treatment, and care-seeking. This finding is unexpected, as one might expect that women who justify violence would be less likely to seek care due to internalized norms and stigma. One possible explanation is that attitudes toward wife beating may act as a proxy for actual exposure to violence; women who normalize this violence may also face more violence [50,51]. This can increase their initial diagnosis for physical injuries. That can lead to their exposure to health services and ultimately diagnosis, care-seeking, and treatment of mental illness as well as physical injury. In coherence with our study, domestic violence was found to be a more significant factor in the diagnosis of mental health illness than others [52–54]. A study about postpartum women in Bangladesh reported that women suffering from physical, sexual, and psychological abuse suffered from significantly higher amounts of postpartum depression than women who didn’t face any violence [51,55].

On the other hand, sociocultural dynamics endorse wife beating in extended family systems or communities where violence is normalized [56,57]. Nevertheless, access to maternal and child services is high in Bangladesh [6]. Although access to maternal and child health (MCH) services is relatively high in Bangladesh, these services do not routinely screen for mental health conditions. However, women who use MCH services may have more interactions with healthcare providers, creating indirect opportunities for the detection and treatment of anxiety and depression. Alternatively, justifying violence may reflect a form of adaptive normalization, where women accept violence but still seek help when mental health symptoms become severe, highlighting a complex interplay between social norms, exposure, and health-seeking behaviors [58,59]. Furthermore, age, division, accounts in bank or financial institutions, and attitude towards wife beating were significant determinants of treatment. Similarly, a study consisting of a rural Australian sample found that older age was significantly associated with delayed treatment of anxiety and depressive disorders [60]. A study in Nepal found that age and administrative division were significant factors in participants’ mental health treatment [61]. Again, age, division, wealth status, working status, attitude towards wife-beating, and internet use were significantly associated with care-seeking. Specifically, employed women may have greater financial resources, autonomy, and social networks, which facilitate access to mental health services [23]. Similarly, internet use can increase awareness of mental health issues, available services, and treatment options, thereby promoting care-seeking behavior [62]. Likewise, in one longitudinal study in Sweden, patients’ age was a significant factor in seeking care [63]. A past study of Bangladesh found that geographical location significantly affected the mental health stigma of people in the country, which further validates our findings [64].

The mental health care situation of the Khulna division presented a striking puzzle. Women had a higher prevalence of diagnosis and a lower prevalence of treatment. It presents a crucial discrepancy in demand versus supply of mental health care for women. Previous studies suggested that it can be caused by structural barriers such as availability, high treatment costs, social barriers, fear, stigmatization, and lack of awareness [64–66]. The pattern observed in Khulna points to geographically specific challenges within the healthcare system, suggesting that even when mental health issues are recognized, there are obstacles preventing women from accessing or completing treatment. Understanding these barriers is essential for interpreting regional disparities and contextualizing mental health care coverage in Bangladesh. It is also important to note that not all diagnosed cases of anxiety and depression require formal clinical or pharmacological treatment. Evidence suggests that individuals with mild to moderate symptoms, as measured by tools such as the GAD-7 and PHQ-9, may benefit from psychosocial support or informal care rather than medical treatment [67,68].

Our study found that middle-class households were more likely to seek care than the richest social class. A study in Nepal also found the same result [40]. The study also found a positive relationship between women’s empowerment and care-seeking for mental disorders. Our study also found a positive relationship between women’s working status and care-seeking for mental disorders, which often leads to women’s empowerment both financially and in decision-making [40,69]. A scoping review also found women’s empowerment incentives as a key to the treatment and care of mental health disorders.[70] In contrast to our study, domestic and intimate partner violence was identified as a barrier to treatment and care-seeking for mental health disorders in several studies [71–73]. Similar to our findings, internet use was found to be an enabling factor for mental health care-seeking and treatment in studies across many countries [43,74,75]. The internet increases awareness of mental health issues, provides information on available services, and allows women to seek guidance privately, thereby reducing barriers related to stigma or limited local resources [76,77].

### Strengths and limitations

It is one of the first studies to explore the situation and factors associated with diagnosis, treatment, and care-seeking among a nationally representative sample of reproductive-aged women. Consequently, it will be more acceptable and more widely generalizable. Moreover, our study separately observed diagnosis, treatment, and care-seeking, providing a precise picture of each indicator. This study used log-binomial regression, which is better suited for communication than logistic regression [34]. In addition to strengths, our study has some pivotal limitations. DHS used a cross-sectional research design, which is not exemplary for causal inferences [78]. Additionally, self-reported data may be subject to recall and social desirability‌‌ biases [79].

## Conclusion and Recommendations

The findings of this study reveal that diagnosis, treatment, and care-seeking for anxiety and depression among reproductive-aged women in Bangladesh remain critically low. A substantial gap exists between diagnosis and treatment, and a large proportion of women rely on informal sources of care rather than formal health services. These patterns highlight significant unmet needs within the mental health care system.

The study findings suggest that structural and systemic barriers play a key role in limiting access to formal mental health services. The reliance on informal care indicates potential issues such as a lack of awareness, stigma, limited availability of trained providers, and weak referral mechanisms within the healthcare system. Additionally, the observed regional disparities, particularly in divisions such as Khulna, indicate uneven distribution of services and access across the country.

Based on these findings, several context-specific recommendations can be made. First, efforts should focus on strengthening the integration of mental health services into existing primary healthcare systems, particularly maternal and reproductive health services. This approach can improve early identification and facilitate timely treatment without requiring entirely new infrastructure.

Second, the substantial gap between diagnosis and treatment highlights the need to improve referral and follow-up systems. Ensuring that women who are diagnosed are effectively linked to appropriate treatment services is critical. This may involve training frontline healthcare providers, including community health workers, to provide basic mental health support and ensure continuity of care.

Third, given the high reliance on informal care, community-based interventions should be prioritized. Engaging community health workers, local leaders, and family members can help reduce stigma and improve awareness about mental health conditions and the importance of seeking formal care.

Fourth, targeted interventions are needed in underserved regions, such as Khulna, where the mismatch between diagnosis and treatment appears more pronounced. Addressing regional inequities in service availability and accessibility should be a key policy priority.

While digital platforms may offer opportunities to support mental health awareness, their applicability remains limited in Bangladesh due to low internet access and urban-rural disparities; therefore, such approaches should be considered supplementary rather than primary strategies.

Finally, future research should further explore the distinction between formal and informal care-seeking pathways to understand better the barriers and facilitators influencing each. Longitudinal studies would also be valuable for better assessing causal relationships and transitions across the mental health care continuum.

## References

[1] World Helath Organization. Anxiety disorders. World Health Organization. https://www.who.int/news-room/fact-sheets/detail/anxiety-disorders 2023. 2024 September 10.

[2] Depressive disorder (depression). WHO. 2023. https://www.who.int/news-room/fact-sheets/detail/depression

[3] Alonso J, Angermeyer MC, Bernert S, Bruffaerts R, Brugha TS, Bryson H, et al. Use of mental health services in Europe: results from the European Study of the Epidemiology of Mental Disorders (ESEMeD) project. Acta Psychiatr Scand Suppl. 2004;(420):47–54. doi: 10.1111/j.1600-0047.2004.00330.x 15128387

[4] Alonso J, Liu Z, Evans-Lacko S, Sadikova E, Sampson N, Chatterji S, et al. Treatment gap for anxiety disorders is global: Results of the World Mental Health Surveys in 21 countries. Depress Anxiety. 2018;35(3):195–208. doi: 10.1002/da.22711 29356216 PMC6008788

[5] Hämäläinen J, Isometsä E, Sihvo S, Pirkola S, Kiviruusu O. Use of health services for major depressive and anxiety disorders in Finland. Depress Anxiety. 2008;25(1):27–37. doi: 10.1002/da.20256 17238158

[6] National Institute of Population Research and Training (NIPORT), I C F. Bangladesh Demographic and Health Survey 2022: Final Report. Dhaka, Bangladesh: National Institute of Population Research and Training (NIPORT). 2024.

[7] Hofmann SG, Smits JAJ. Cognitive-behavioral therapy for adult anxiety disorders: a meta-analysis of randomized placebo-controlled trials. J Clin Psychiatry. 2008;69(4):621–32. doi: 10.4088/jcp.v69n0415 18363421 PMC2409267

[8] Bandelow B, Michaelis S, Wedekind D. Treatment of anxiety disorders. Dialogues Clin Neurosci. 2017;19(2):93–107. doi: 10.31887/DCNS.2017.19.2/bbandelow 28867934 PMC5573566

[9] Centers for Disease Control and Prevention. Mental Health Conditions: Depression and Anxiety. CDC. https://www.cdc.gov/tobacco/campaign/tips/diseases/depression-anxiety.html. 2023. 2025 February 5.

[10] Barlow DH. Anxiety and Its Disorders: The Nature and Treatment of Anxiety and Panic. 2nd ed. New York: Guilford Publications. 2004.

[11] Kasper S. Anxiety disorders: under-diagnosed and insufficiently treated. Int J Psychiatry Clin Pract. 2006;10 Suppl 1:3–9. doi: 10.1080/13651500600552297 24931537

[12] Schonfeld WH, Verboncoeur CJ, Fifer SK, Lipschutz RC, Lubeck DP, Buesching DP. The functioning and well-being of patients with unrecognized anxiety disorders and major depressive disorder. J Affect Disord. 1997;43(2):105–19. doi: 10.1016/s0165-0327(96)01416-4 9165380

[13] Scott AJ, Bisby MA, Heriseanu AI, Hathway T, Karin E, Gandy M, et al. Understanding the untreated course of anxiety disorders in treatment-seeking samples: A systematic review and meta-analysis. J Anxiety Disord. 2022;89:102590. doi: 10.1016/j.janxdis.2022.102590 35689850

[14] Ogunyemi D, Jovanovski A, Liu J, Friedman P, Sugiyama N, Creps J. The Contribution of Untreated and Treated Anxiety and Depression to Prenatal, Intrapartum, and Neonatal Outcomes. Am J Perinatol Reports. 2018;08:e146–57. doi: 10.1055/s-0038-1661379 PMC6039295 29998037

[15] Carlson K, Mughal S, Azhar Y, Siddiqui W. Perinatal Depression. 2025 StatPearls. Treasure Island (FL): StatPearls Publishing; 30085612

[16] Sifat MS, Tasnim N, Hoque N, Saperstein S, Shin RQ, Feldman R, et al. Motivations and barriers for clinical mental health help-seeking in Bangladeshi university students: a cross-sectional study. Glob Ment Health (Camb). 2022;9:211–20. doi: 10.1017/gmh.2022.24 36618754 PMC9806995

[17] Dutta GK, Sarker B, Ahamed H, Bhattacharyya DS, Rahman M, Biswas T. Mental healthcare-seeking behavior during the perinatal period among rural women in Bangladesh. 2021. doi: 10.21203/rs.3.rs-459178/v1 PMC8900444 35255914

[18] Rahman NAS, Mustafa M, Tabassum T, Simu S, Gupta M, Afrin S. Health-seeking behavior and anxiety of cancer patients in Bangladesh during the COVID-19 pandemic: A cross-sectional study. 2024. doi: 10.1101/2024.08.19.24312282 PMC12633963 41280602

[19] Huque R, Azad A, Islam K, Ahmed H, Amin M. Care Seeking Behavior and Treatment Gap for Mental Health Conditions in Bangladesh: Evidence from the National Mental Health Survey 2019. 2023. doi: 10.21203/rs.3.rs-3678497/v1

[20] Chowdhury H, Islam M, Morshed NM, Algin S. The pattern of admission, discharge, and outcome at a private psychiatry hospital in Dhaka, Bangladesh: A retrospective study. J Psychiatry Psychiatr Disord. 2023;7:167–73. doi: 10.26502/jppd.2572-519X0196

[21] Ahmed T, Luthfa ST, Chaahat AH, Kanchon MR, Sharif AB. Determinants of early mental health help-seeking among women in Bangladesh: A nationally representative bootstrapped regression analysis. PLOS Ment Health. 2025;2(9):e0000420. doi: 10.1371/journal.pmen.0000420 41662058 PMC12798417

[22] Walter H, Craig ME, Ali M, Faruque S, Saha S. Seeking support: insights into women’s mental health help-seeking behavior in Bangladesh. Front Glob Womens Health. 2025;6:1679141. doi: 10.3389/fgwh.2025.1679141 41268467 PMC12627062

[23] Rafi MA, Anika US, Hasan MT, Hossain MG. Association between women’s empowerment and mental health help-seeking behaviour in Bangladesh: findings from a nationally representative survey. BMJ Open. 2025;15(9):e099770. doi: 10.1136/bmjopen-2025-099770 40953863 PMC12434786

[24] Raza S, Banik R, Noor STA, Sayeed A, Saha A, Jahan E, et al. Anxiety and depression among reproductive-aged women in Bangladesh: burden, determinants, and care-seeking practices based on a nationally representative demographic and health survey. Arch Womens Ment Health. 2025;28(5):1125–41. doi: 10.1007/s00737-025-01564-3 39964560 PMC12436553

[25] Wang PS, Aguilar-Gaxiola S, Alonso J, Angermeyer MC, Borges G, Bromet EJ, et al. Use of mental health services for anxiety, mood, and substance disorders in 17 countries in the WHO world mental health surveys. Lancet. 2007;370(9590):841–50. doi: 10.1016/S0140-6736(07)61414-7 17826169 PMC2847360

[26] Rickwood D, Deane F, Wilson C, Ciarrochi J. Young people’s help-seeking for mental health problems. Adv Ment Heal. 2005;4:218. doi: 10.5172/jamh.4.3.218

[27] Gulliver A, Griffiths KM, Christensen H. Perceived barriers and facilitators to mental health help-seeking in young people: a systematic review. BMC Psychiatry. 2010;10:113. doi: 10.1186/1471-244X-10-113 21192795 PMC3022639

[28] Graham A, Hasking P, Brooker J, Clarke D, Meadows G. Mental health service use among those with depression: an exploration using Andersen’s Behavioral Model of Health Service Use. J Affect Disord. 2017;208:170–6. doi: 10.1016/j.jad.2016.08.074 27788380

[29] Talty FT, Roberts ME, Dang C, Clewley DJ, Horn ME. Using a behavioral model to identify factors associated with choice of provider for neck and low back pain: A systematic review. Musculoskelet Sci Pract. 2020;49:102223. doi: 10.1016/j.msksp.2020.102223 32763791

[30] Xin Y, Ren X. Determinants of province-based health service utilization according to Andersen’ s Behavioral Model: a population-based spatial panel modeling study. BMC Public Health. 2023;23(1):985. doi: 10.1186/s12889-023-15885-4 37237347 PMC10224305

[31] ALBashtawy M. Application and use of Andersen’s behavioral model as theoretical framework: A systematic literature review from 2012–2021. 2023. 10.18502/ijph.v52i7.13236 PMC10430393 37593505

[32] Zou S, Qi X, Marshall K, Bhura M, Takesue R, Tang K. Understanding the context of healthcare utilisation for children under-five with diarrhoea in the DRC: based on Andersen behavioural model. BMC Health Serv Res. 2022;22(1):144. doi: 10.1186/s12913-022-07530-4 35120503 PMC8815172

[33] Rao JNK, Scott AJ. The analysis of categorical data from complex sample surveys: Chi-squared tests for goodness of fit and independence in two-way tables. J Am Stat Assoc. 1981;76:221–30. doi: 10.2307/2287815

[34] Williamson T, Eliasziw M, Fick GH. Log-binomial models: exploring failed convergence. Emerg Themes Epidemiol. 2013;10(1):14. doi: 10.1186/1742-7622-10-14 24330636 PMC3909339

[35] Amin MT, Ara T, Pal B, Ferdous Z, Esha SN, Patwary H, et al. Prevalence and correlates of anxiety and depression among ever-married reproductive-aged women in Bangladesh: national-level insights from the 2022 Bangladesh Demographic and Health Survey. BMC Public Health. 2025;25(1):1143. doi: 10.1186/s12889-025-22228-y 40133877 PMC11938691

[36] Haque MR, Fazle Rabbi AM, Araf F, Rahman MM. Prevalence of anxiety and depression among married women in Bangladesh: An analysis of nationally representative survey. PLOS Ment Health. 2025;2(7):e0000387. doi: 10.1371/journal.pmen.0000387 41662041 PMC12798323

[37] Barbui C. The WHO World Mental Health Report 2022: a new standard of care is emerging. Mol Psychiatry. 2022;28. doi: 10.1038/s41380-022-01788-0 36123421

[38] Kirkbride JB, Anglin DM, Colman I, Dykxhoorn J, Jones PB, Patalay P, et al. The social determinants of mental health and disorder: evidence, prevention and recommendations. World Psychiatry. 2024;23(1):58–90. doi: 10.1002/wps.21160 38214615 PMC10786006

[39] Gold KJ, Andrew LB, Goldman EB, Schwenk TL. “I would never want to have a mental health diagnosis on my record”: A survey of female physicians on mental health diagnosis, treatment, and reporting. Gen Hosp Psychiatry. 2016;43:51–7. doi: 10.1016/j.genhosppsych.2016.09.004 27796258

[40] Shawon MSR, Hossain FB, Ahmed R, Poly IJ, Hasan M, Rahman MR. Role of women empowerment on mental health problems and care-seeking behavior among married women in Nepal: secondary analysis of nationally representative data. Arch Womens Ment Health. 2024;27(4):527–36. doi: 10.1007/s00737-024-01433-5 38315185 PMC11230993

[41] Azale T, Fekadu A, Hanlon C. Treatment gap and help-seeking for postpartum depression in a rural African setting. BMC Psychiatry. 2016;16:196. doi: 10.1186/s12888-016-0892-8 27287387 PMC4901434

[42] Qiu P, Caine ED, Hou F, Cerulli C, Wittink MN. Depression as seen through the eyes of rural Chinese women: Implications for help-seeking and the future of mental health care in China. J Affect Disord. 2018;227:38–47. doi: 10.1016/j.jad.2017.10.016 29053974 PMC5805647

[43] Schwank SE, Andersson E, Wickberg B, Fu S-C, Ding Y, Lindgren H. Care-seeking behavior and disclosure on self-reported mental health among young women in urban Shanghai, China. Health Psychol Open. 2020;7(1):2055102919897382. doi: 10.1177/2055102919897382 32082605 PMC7005976

[44] World Health Organization. WHO Special Initiative for Mental Health: Nepal. https://www.who.int/about/accountability/results/who-results-report-2020-mtr/country-story/2022/improving-access-to-mental-health-services-by-integrating-them-into-general-health-services-in-nepal?utm_source=chatgpt.com 2026 March 26.

[45] Upadhaya N, Regmi U, Gurung D, Luitel NP, Petersen I, Jordans MJD, et al. Mental health and psychosocial support services in primary health care in Nepal: perceived facilitating factors, barriers and strategies for improvement. BMC Psychiatry. 2020;20(1):64. doi: 10.1186/s12888-020-2476-x 32054462 PMC7020582

[46] Xu T, Ni R, Wu H, Xu F, Song S, Yuan X, et al. Analysis of psychiatrists’ internet service patterns: a cross-sectional study from China’s largest online mental health platform. Front Psychiatry. 2025;16:1598574. doi: 10.3389/fpsyt.2025.1598574 40642412 PMC12241070

[47] Zhang X, Lewis S, Firth J, Chen X, Bucci S. Digital mental health in China: a systematic review. Psychol Med. 2021;51(15):2552–70. doi: 10.1017/S0033291721003731 34581263 PMC8579156

[48] Koly KN, Saba J, Muzaffar R, Modasser RB, M TH, Colon-Cabrera D, et al. Exploring the potential of delivering mental health care services using digital technologies in Bangladesh: A qualitative analysis. Internet Interv. 2022;29:100544. doi: 10.1016/j.invent.2022.100544 35615404 PMC9125629

[49] Siegel AM, Mathews SB. Diagnosis and Treatment of Anxiety in the Aging Woman. Curr Psychiatry Rep. 2015;17(12):93. doi: 10.1007/s11920-015-0636-3 26458819

[50] Uthman OA, Lawoko S, Moradi T. Factors associated with attitudes towards intimate partner violence against women: A comparative analysis of 17 sub-saharan countries. Social Work and Community Practice. 2016;:275–95. 10.1186/1472-698X-9-14 PMC2718859 19619299

[51] Antai D. Controlling behavior, power relations within intimate relationships and intimate partner physical and sexual violence against women in Nigeria. BMC Public Health. 2011;11:511. doi: 10.1186/1471-2458-11-511 21714854 PMC3161889

[52] Tinglöf S, Högberg U, Lundell I, Svanberg A. Exposure to violence among women with unwanted pregnancies and the association with post-traumatic stress disorder, symptoms of anxiety and depression. Sex Reprod Healthc. 2014;6. doi: 10.1016/j.srhc.2014.08.003 25998870

[53] Ludermir AB, Schraiber LB, D’Oliveira AFPL, França-Junior I, Jansen HA. Violence against women by their intimate partner and common mental disorders. Soc Sci Med. 2008;66(4):1008–18. doi: 10.1016/j.socscimed.2007.10.021 18178299

[54] Ahmadabadi Z, Najman JM, Williams GM, Clavarino AM, d’Abbs P, Tran N. Intimate partner violence and subsequent depression and anxiety disorders. Soc Psychiatry Psychiatr Epidemiol. 2020;55(5):611–20. doi: 10.1007/s00127-019-01828-1 31912167

[55] Islam MJ, Broidy L, Baird K, Mazerolle P. Intimate partner violence around the time of pregnancy and postpartum depression: The experience of women of Bangladesh. PLoS One. 2017;12(5):e0176211. doi: 10.1371/journal.pone.0176211 28472056 PMC5417480

[56] Kashinath G, Parasar A. Domestic violence during covid-19 pandemic: The case for Indian women. 2022. 10.1002/casp.2501 PMC8014493 33821118

[57] Raj A, Livramento K, Santana M, Gupta J, Silverman J. Victims of intimate partner violence more likely to report abuse from in-laws. Violence Against Women. 2006;12:936–49. doi: 10.1177/1077801206292935 16957174

[58] Yount KM, Li L. Women’s “Justification” of Domestic Violence in Egypt. J of Marriage and Family. 2009;71(5):1125–40. doi: 10.1111/j.1741-3737.2009.00659.x

[59] Sarkar NN. The impact of intimate partner violence on women’s reproductive health and pregnancy outcome. J Obstet Gynaecol. 2008;28(3):266–71. doi: 10.1080/01443610802042415 18569465

[60] Green AC, Hunt C, Stain HJ. The delay between symptom onset and seeking professional treatment for anxiety and depressive disorders in a rural Australian sample. Soc Psychiatry Psychiatr Epidemiol. 2012;47(9):1475–87. doi: 10.1007/s00127-011-0453-x 22116199

[61] Sharma TP. Mental Health Treatment Patterns for Anxiety and Depression among Women in Nepal. Asian J Pop Sci. 2025;4(1):60–72. doi: 10.3126/ajps.v4i1.73905

[62] Liu L, Pang Y, Jia X. Online health information seeking behavior: A systematic review. Healthcare. 2022;9. doi: 10.3390/healthcare9121740 PMC8701665 34946466

[63] Wallerblad A, Möller J, Forsell Y. Care-Seeking Pattern among Persons with Depression and Anxiety: A Population-Based Study in Sweden. Int J Family Med. 2012;2012:895425. doi: 10.1155/2012/895425 22655197 PMC3357962

[64] Faruk MO, Khan AH, Chowdhury KUA, Jahan S, Sarker DC, Colucci E, et al. Mental illness stigma in Bangladesh: Findings from a cross-sectional survey. Glob Ment Health (Camb). 2023;10:e59. doi: 10.1017/gmh.2023.56 37854431 PMC10579681

[65] Thornicroft G. Stigma and discrimination limit access to mental health care. Epidemiol Psichiatr Soc. 2008;17(1):14–9. doi: 10.1017/s1121189x00002621 18444452

[66] Gao Y, Burns R, Leach L, Chilver MR, Butterworth P. Examining the mental health services among people with mental disorders: a literature review. BMC Psychiatry. 2024;24(1):568. doi: 10.1186/s12888-024-05965-z 39164690 PMC11334396

[67] Kroenke K, Spitzer RL, Williams JB. The PHQ-9: validity of a brief depression severity measure. J Gen Intern Med. 2001;16(9):606–13. doi: 10.1046/j.1525-1497.2001.016009606.x 11556941 PMC1495268

[68] Spitzer RL, Kroenke K, Williams JBW, Löwe B. A brief measure for assessing generalized anxiety disorder: the GAD-7. Arch Intern Med. 2006;166(10):1092–7. doi: 10.1001/archinte.166.10.1092 16717171

[69] Riaz S, Pervaiz Z. The impact of women’s education and employment on their empowerment: an empirical evidence from household level survey. Qual Quant. 2018;52. doi: 10.1007/s11135-018-0713-x

[70] Bandara NA, Al-Anzi SMF, Zhdanova A, Hirani S. Women’s Empowerment and Mental Health: A Scoping Review. Women. 2024;4(3):277–89. doi: 10.3390/women4030021

[71] Abrahams Z, Boisits S, Schneider M, Honikman S, Lund C. Facilitators and barriers to detection and treatment of depression, anxiety and experiences of domestic violence in pregnant women. Sci Rep. 2023;13(1):12457. doi: 10.1038/s41598-023-36150-z 37528133 PMC10394005

[72] Ferrari G, Agnew-Davies R, Bailey J, Howard L, Howarth E, Peters TJ, et al. Domestic violence and mental health: a cross-sectional survey of women seeking help from domestic violence support services. Glob Health Action. 2014;7:25519. doi: 10.3402/gha.v7.25519 25319597 PMC4199331

[73] Grigaitė U, Azeredo-Lopes S, Žeimė E, Yamin P, Heitmayer M, Aluh D. Use of mental health services by survivors of psychological intimate partner violence in Lithuania. 2024. 10.1093/pubmed/fdae015 38336363

[74] Van Ameringen M, Simpson W, Patterson B, Turna J. Internet screening for anxiety disorders: Treatment-seeking outcomes in a three-month follow-up study. Psychiatry Res. 2015;230(2):689–94. doi: 10.1016/j.psychres.2015.10.031 26553144

[75] Van Meter A, Birnbaum M, Rizvi A, Kane J. Online help-seeking prior to diagnosis: Can web-based resources reduce the duration of untreated mood disorders in young people?. J Affect Disord. 2019;252. doi: 10.1016/j.jad.2019.04.019 PMC6529208 30981056

[76] Yuen EK, Gangi CE, Barakat K, Harrison F. College students’ utilization of the Internet to search for mental health information: Effects on mental health literacy, stigma, and help-seeking. J Am Coll Health. 2025;73(3):961–71. doi: 10.1080/07448481.2024.2404948 39303085

[77] Powell J, Clarke A. Internet information-seeking in mental health: population survey. Br J Psychiatry. 2006;189:273–7. doi: 10.1192/bjp.bp.105.017319 16946364

[78] Sedgwick P. Cross sectional studies: advantages and disadvantages. BMJ. 2014;348(mar26 2):g2276–g2276. doi: 10.1136/bmj.g2276 25134102

[79] Rosenman R, Tennekoon V, Hill LG. Measuring bias in self-reported data. Int J Behav Healthc Res. 2011;2(4):320–32. doi: 10.1504/IJBHR.2011.043414 25383095 PMC4224297

